# Targeting cellular metabolism to reduce head and neck cancer growth

**DOI:** 10.1038/s41598-019-41523-4

**Published:** 2019-03-21

**Authors:** Jian Yang, Yuqi Guo, Wonkyu Seo, Ruohan Zhang, Cuijie Lu, Yaoyu Wang, Liang Luo, Bidisha Paul, Wenbo Yan, Deepak Saxena, Xin Li

**Affiliations:** 1 Department of Basic Science and Craniofacial Biology, New York University College of Dentistry, New York, NY 10010 USA; 2 Department of Surgery, New York University Langone Medical Center, New York, NY 10016 USA; 3 Department of Urology, New York University Langone Medical Center, New York, NY 10016 USA; 4 Perlmutter Cancer Institute, New York University Langone Medical Center, New York, NY 10016 USA

## Abstract

Head and neck squamous cell carcinoma (HNSCC) presents a major public health concern because of delayed diagnosis and poor prognosis. Malignant cells often reprogram their metabolism in order to promote their survival and proliferation. Aberrant glutaminase 1 (GLS1) expression enables malignant cells to undergo increased glutaminolysis and utilization of glutamine as an alternative nutrient. In this study, we found a significantly elevated GLS1 expression in HNSCC, and patients with high expression levels of GLS1 experienced shorter disease-free periods after therapy. We hypothesized that the GLS1 selective inhibitor, bis-2-(5-phenylacetamido-1,3,4-thiadiazol-2-yl)ethyl sulfide (BPTES), which curtails cells’ glutamine consumption, may inhibit HNSCC cell growth. Our results support the idea that BPTES inhibits HNSCC growth by inducing apoptosis and cell cycle arrest. Considering that metformin can reduce glucose consumption, we speculated that metformin would enhance the anti-neoplasia effect of BPTES by suppressing malignant cells’ glucose utilization. The combination of both compounds exhibited an additive inhibitory effect on cancer cell survival and proliferation. All of our data suggest that GLS1 is a promising therapeutic target for HNSCC treatment. Combining BPTES with metformin might achieve improved anti-cancer effects in HNSSC, which sheds light on using novel therapeutic strategies by dually targeting cellular metabolism.

## Introduction

Head and neck cancer is the sixth most common cancer worldwide, accounting for around 2.5% of new cancer cases and 1.9% of deaths annually^1^. The incidence rate of oral cancer is highly related to detrimental oral-associated behaviors, including smoking and alcohol consumption in addition to exposure to the human papillomavirus^2^. Smoking and alcohol have been shown to be involved in an altered metabolic state and carcinogenesis^3,4^. Metabolic reprogramming is a hallmark of all types of cancer. Common reprogramming includes the Warburg effect, which produces energy through glycolysis, and utilization of alternative energy resources such as glutamine through glutaminolysis. Thus, small molecular inhibitors that target tumor-specific metabolism represent promising anti-cancer approaches^5^.

GLS1 is a key enzyme that is required in the first step of glutamine metabolism. It acts through catalyzing the conversion of glutamine into glutamate and ammonia. Glutamate is further metabolized by glutamate dehydrogenase or transaminases to α-ketoglutarate and then oxidized in the tricarboxylic acid (TCA) cycle^6^. The allosteric glutaminase-selective inhibitor, bis-2-(5-phenylacetamido-1,3,4-thiadiazol-2-yl)ethyl sulfide (BPTES), specifically inhibits glutamine metabolism with minimal off-target effects^7^. BPTES has been shown to significantly prolong the survival of animals with MYC-induced hepatocellular carcinoma^8^. In addition, BPTES suppresses the von Hippel-Lindau-deficient renal cancers^9^.

Metformin is a biguanide-derived molecule from the French lilac and is the most commonly prescribed oral anti-diabetic drug for the treatment of type 2 diabetes mellitus^10^. Metformin is also used to combat polycystic ovarian syndrome, metabolic syndrome, and obesity^11,12^. Since it was approved by the Food and Drug Administration in 1994, metformin has been extensively researched for its anti-neoplastic effects. There has been extensive research linking metformin to a lower incidence of mortality in liver, colorectal, pancreatic, stomach, and esophageal cancer^13^. The phosphoinositide 3 kinase (PI3K), protein kinase B (Akt), and the mammalian target of rapamycin (mTOR) complex 1 (mTORC1) signaling pathways, which have been implicated in HNSCC progression^14–16^ and have been shown to be inhibited by metformin^17^. Our previous study revealed that metformin can regulate the cell cycle and apoptosis by suppressing prostate cancer growth via regulation of oncogene c-Myc^18^ and tumor suppressor miRNA-708^19^. These results indicate that metformin is capable of dampening multiple oncogenic signals that will cause tumor growth suppression. Of note, metformin also affects tumor growth by down-regulating glucose consumption in tumor cells^20^. Therefore, we hypothesized that BPTES and metformin may produce a promising additive effect by concurrently targeting both glutamine and glucose metabolic pathways.

In this study, we evaluated the expression of GLS1 and showed its negative correlation with disease-free periods in patients with HNSCC. We further investigated the BPTES and metformin combination’s anti-tumorigenic potential and their combined effects on apoptosis and cell-cycle arrest in HNSCC cell lines.

## Results

### Glulaminase 1 (GLS1) is highly expressed in HNSCC cells

**Figure 1:**
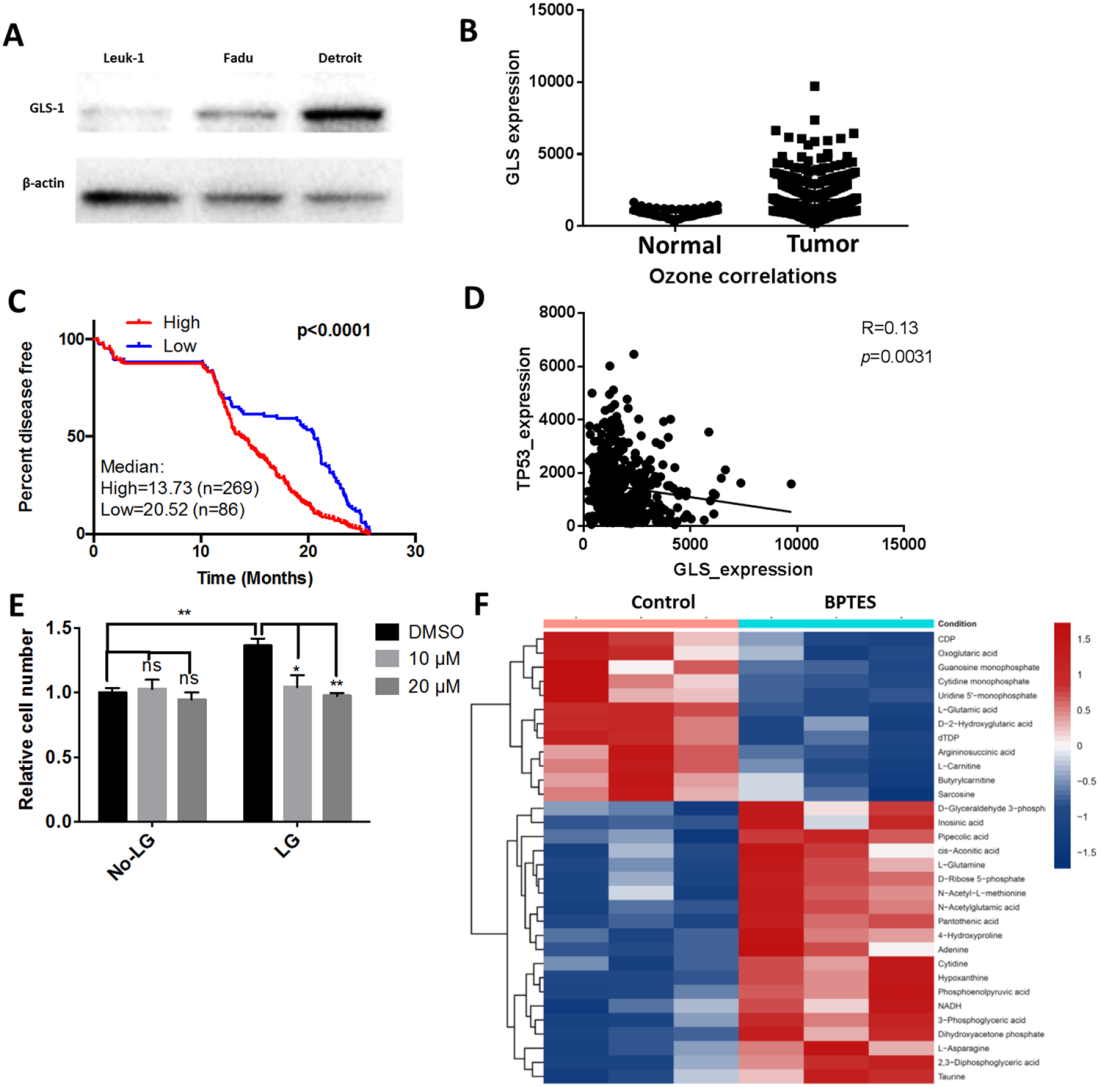
GLS1 is highly expressed in HNSCC. (**A**) GLS1 expression level in Leuk-1, FaDu and Detroit cell lines were determined by western blotting. β-actin was used as internal control (**B**) GLS1 expression level in HNSCC patients and normal controls were evaluated using a TCGA cohort. (**C**) Kaplan-Meier survival curve was generated to show the percentage of disease-free patients of GLS-high and GLS-low subgroups after receiving therapy. (**D**) Correlation between TP53 and GLS in the TCGA dataset was analyzed Prism Graphpad software. (**E**) 10,000 of Detroit cells were seeded in 96-well plate and cultured in low glucose (5 mM) DMEM medium supplement with or without L-glutamine (2 mM). Cells were treated with BPTES at indicated concentration for 48 hours. Cell viability was determined using MTT assay. ^**^p < 0.01, ^****^p < 0.0001. (**F**) Metabolomics assay was performed using FaDu cell samples treated with control or 10 µM BPETS for 72 hours. DMSO was used as control. Heat-map represents indicated metabolites changes of both groups (N = 3).

In order to evaluate GLS1 expression levels in HNSCC cells, we performed western blotting using the squamous cancer and pharyngeal carcinoma cell lines, FaDu and Detroit 562, respectively. The pre-malignant oral leukoplakia cell line MSK Leuk-1 was used as a control for these experiments. GLS1 expression was dramatically elevated in HNSCC cell lines (Fig. 1A). We also analyzed GLS1 expression in a cohort of human patients consisting of 44 healthy samples and 522 tumor samples from The Cancer Genome Atlas (TCGA) database. We found that the mean GLS1 expression level in tumor samples was twice as high as in healthy controls (Fig. 1B). When comparing the GLS-low to the GLS-high subgroup, a Kaplan-Meier curve revealed that patients with low GLS expression lived significantly longer with disease-free periods than did the GLS-high subgroup (Fig. 1C). Further analysis revealed that GLS expression was negatively associated with tumor protein (TP)53 (Fig. 1D), indicating that targeting GLS may improve the overall survival of TP53 mutant patients whose prognosis is poorer than the wild-type TP53 subset^21^. These data strongly support the relevance of GLS in HNSCC.

### BPTES and metformin inhibit the cell growth of HNSCC cells

We treated the cells with BPTES, a selective GLS1 inhibitor, in order to further examine the role of glutamine metabolism in the growth of HNSCC cells. The cell viability assay using 3-(4,5-dimethylthiazol-2-yl)-2,5-diphenyltetrazolium bromide (MTT) showed that BPTES effectively inhibited the growth of Detroit 562 cells grown in the presence of L-glutamine but had little effect when used in the L-glutamine-free medium (Fig. 1E). As expected, metabolomics analysis revealed that BPETS decreased the levels of downstream metabolites, including glutamic and oxoglutaric acids, along the glutamine metabolism pathway (Fig. 1F). Consistently, real-time polymerase chain reaction (qPCR) results showed that that members of the pyruvate dehydrogenase kinases (PDKs) family are decreased following the treatment of BPTES (Supplementary Fig. 1).

**Figure 2:**
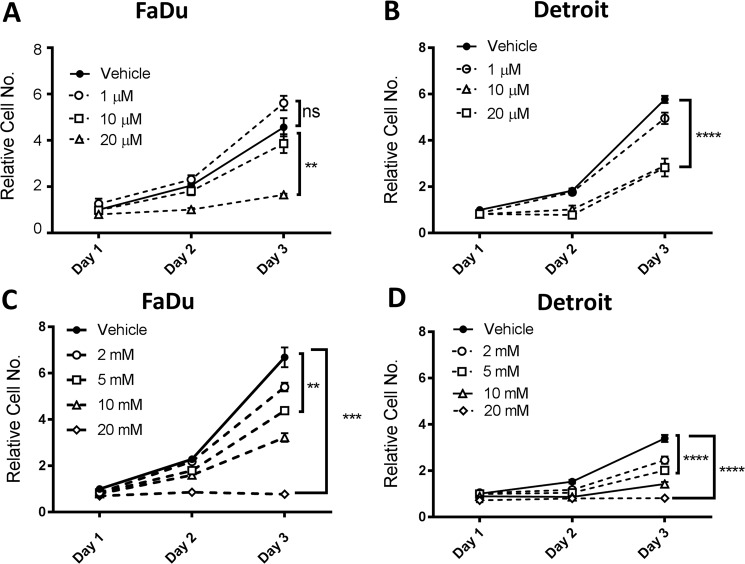
BPTES and metformin suppresses the growth of HNSCC. FaDu and Detroit 562 cell lines were used to assess the effects of BPTES and metformin at indicated concentrations. For both cell lines, 10,000 cells were seeded in a 96-well plate and treated with indicated concentrations of BPTES or metformin for 24 hours, 48 hours and 72 hours. The relative live cell numbers were indicated by the absorbance readings after crystal violet staining. (**A**) FaDu cells treated with Vehicle (DMSO) or BPTES; (**B**) FaDu cells treated with vehicle (PBS) or metformin; (**C**) Detroit 562 cells treated with Vehicle (DMSO) or BPTES; (**D**) Detroit 562 cells treated with vehicle (PBS) or metformin. ^**^p < 0.01, ^***^p < 0.001, ^****^p < 0.0001.

Growth of HNSCC cells was assessed in the presence of a glutaminase-selective inhibitor, BPTES. Several concentrations ranging from 1 to 20 µM were selected according to a previous report^22^ and assessed. The results showed that BPTES inhibited the growth of tumor cells in a dose-dependent fashion in both FaDu and Detroit 562 cells (Fig. 2A,B). Additionally, we confirmed that metformin suppressed cell growth in both Detroit 562 and FaDu cell lines in a dose-dependent manner (Fig. 2C,D).

### BPTES and metformin induces the apoptosis and cell cycle arrest of HNSCC cells

**Figure 3:**
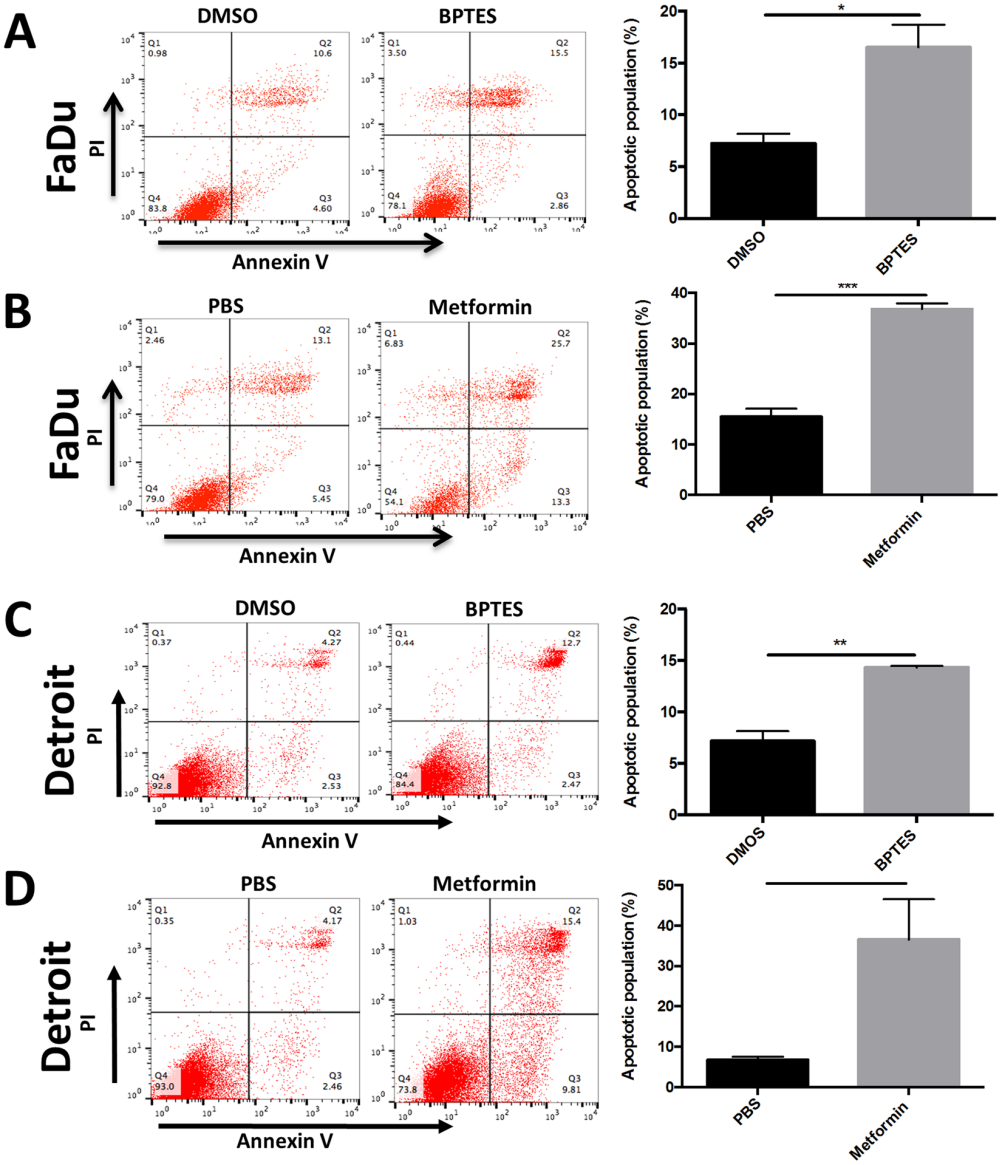
BPTES and metformin induces the apoptosis of HNSCC. Equal number (0.5 × 10^6^) of (**A**,**B**) FaDu or (**C**,**D**) Detroit 562 cells were seeded in 6-well plate and treated with BPTES (20 μM) or metformin (10 mM) for 48 hours. The apoptotic cell population in each well was determined by flow cytometry analysis safter propidium iodide and Annexin V double staining. ^*^p < 0.05, ^**^p < 0.01, ^***^p < 0.001,

In order to further explore the cellular mechanism of BPTES and metformin on inhibition of HNSCC cell growth *in vitro*, we evaluated the level of apoptosis in FaDu cells treated with either BPTES or metformin. The cells were stained with necrotisis and apoptosis dyes (propidium iodide [PI] and annexin V, respectively) followed by flow cytometry analysis in order to quantify the apoptotic and necrotic cell populations. As expected, both BPTES and metformin were capable of inducing apoptosis and necrosis in FaDu and Detroit 562 cell lines. In FaDu cells, treatment with 20 µM BPTES induced a 2-fold increase in the apoptotic cell population (Fig. 3A). Similar effects were observed in FaDu cells treated with 10 mM metformin (Fig. 3B) and Detroit 562 cells treated with 20 µM BPTES (Fig. 3C). Detroit 562 cell apoptosis was stimulated about 5-fold by metformin (Fig. 3D).

**Figure 4:**
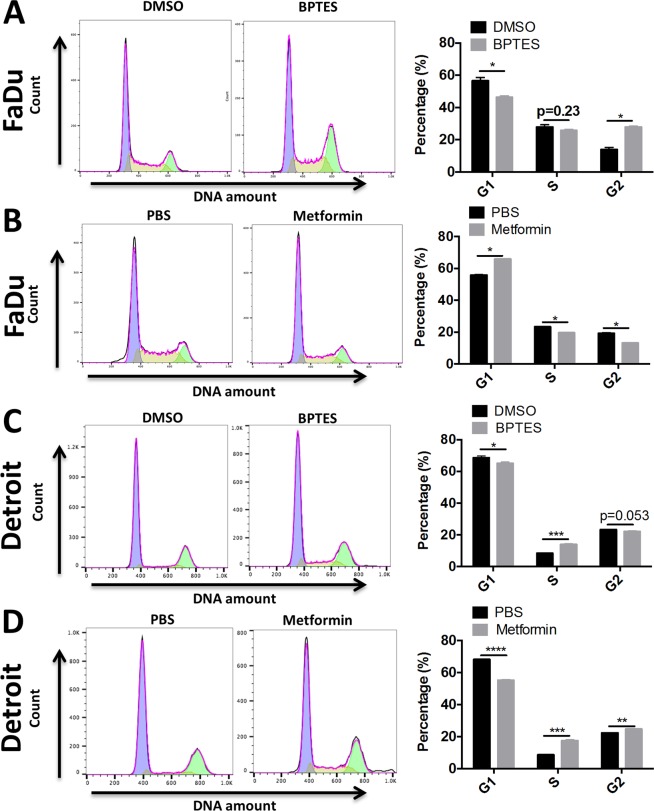
BPTES and metformin induces the cell cycle arrest of FaDu and Detroit cell line. Half-million (**A**,**B**) FaDu or (**C**,**D**) Detroit cells were seeded in 6-well plate and treated with BPTES (20 μM) or metformin (10 mM) for 48 hours. The cell population in each cell cycle phase was determined by flow cytometry analysis after propidium iodide staining. ^*^p < 0.05, ^**^p < 0.01, ^***^p ≤ 0.001, ^****^p < 0.0001.

Further, we performed cell cycle analysis in BPTES- or metformin-treated cancer cells. The DNA content in the G1, S, and G2 phases of cells was measured using flow cytometry after PI staining. Consistent with the delayed cell growth demonstrated by total cell number, flow cytometry showed that BPTES caused cell cycle arrest in the G2 phase in FaDu cells (Fig. 4A), while metformin mainly arrested FaDu cells in the G1 phase (Fig. 4B). In contrast, BPTES caused cell cycle arrest in the S phase in Detroit 562 cells (Fig. 4C), while metformin caused Detroit 562 cell arrest in the S and G2 phases (Fig. 4D).

Collectively, these data justify further exploration of the BPTES and metformin combination’s joint actions on suppressing HNSCC cell growth.

### Combined effect of BPTES and metformin

**Figure 5:**
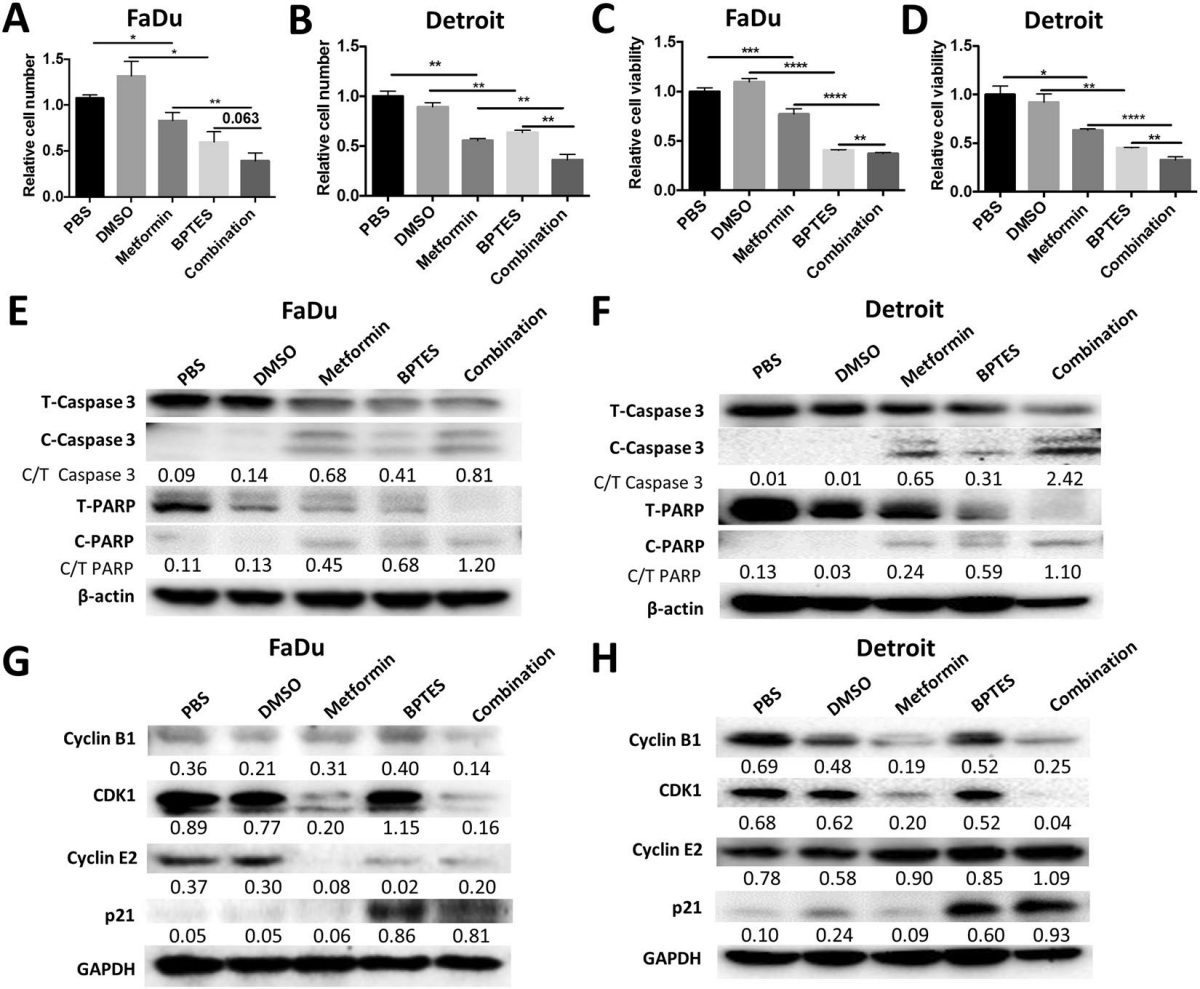
Combined treatment of BPTES and metformin additively inhibits the cell viability of HNSCC. 10,000 of FaDu or Detroit cells were seeded in 96-well plate and treated with BPTES (20 μM) or metformin (10 mM) or the combination for 48 hours. The cell numbers of FaDu (**A**) and Detroit (**B**) cell lines were determined by crystal violet staining. The viability of FaDu (**C**) and Detroit (**D**) cells was determined by MTT assay. Apoptotic markers of FaDu (**E**) and Detroit (**F**) cells t were determined by western blotting. β-actin was used as internal control. The ratio of cleaved Caspase 3 to total Casepase 3 (C/T Caspase 3), or cleaved PARP to total PARP (C/T PARP) was displayed under the band of indicated treatments. Cell cycle regulatory proteins in FaDu (**G**) and Detroit (**H**) cells were determined by western blotting with GAPDH used as internal control. The ratio of indicated protein to internal control was displayed under each band. ^*^p < 0.05, ^**^p < 0.01, ^***^p < 0.001, ^****^p < 0.0001.

Both BPTES and metformin suppressed HNSCC cell growth. Importantly, previous data have indicated that these two agents can initiate apoptosis and target different stages of cell cycle arrest. In order to test the effects of combining BPTES and metformin, crystal violet staining was performed to assess the relative cell numbers after 48 h of treatment. Compared to treatment with a single agent, the combined application of BPTES and metformin exhibited a stronger inhibitory effect on cell growth (Fig. 5A,B). An MTT assay and western blotting experiment also supported the additive effects of the BPTES and metformin combination on cell viability and apoptosis (Fig. 5C–F, respectively). In addition, metformin reduced the cell cycle regulatory proteins, cyclin E2 and the CDK1/cyclin B1 complex. In contrast to metformin, BPTES stimulated the expression of the cell cycle inhibitor, p21. Combined treatment resulted in protein expression patterns reflecting the additive effects of BPTES and metformin treatment (Fig. 5G–H).

**Figure 6:**
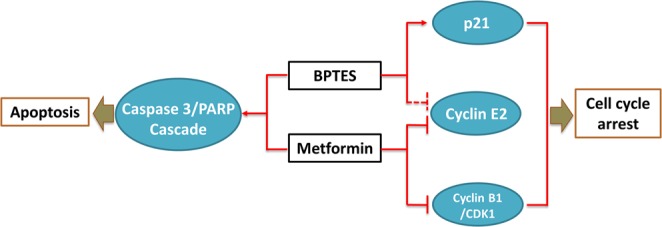
Schematic model depicting the regulatory effect of BPTES and metformin on cell cycle and apoptosis pathways in HNSCC. BPTES reduces the expression of cyclin E2 (in Fadu cells only) and promotes the expression of p21 to induce G2-phase arrest. Metformin decreases CDK1/Cyclin B1 expression and almost totally eliminates Cyclin E2 expression to induce G1-phase arrest. Both BPTES and metformin are able to initiate apoptosis by inducing the cleavage of Caspase 3/PRAP cascade.

In summary, BPTES and metformin most likely target different molecules/pathways that regulate cell metabolism and growth (Fig. 6). BPTES reduces cyclin E2 expression and promotes p21 expression, which will then induce G2-phase arrest. Metformin decreases CDK1/cyclin B1 expression and almost completely eliminates cyclin E2 expression, which will then induce G1-phase arrest. Both BPTES and metformin are capable of inducing apoptosis by stimulating the cleavage of caspase 3.

## Discussion

Our study described GLS1 over-expression in HNSCC and demonstrated that targeting glutaminase activity with BPTES effectively reduced HNSCC growth. Furthermore, we showed that the combined application of BPTES and metformin, which hindered glutamine and glucose consumption, achieved additive suppression of HNSCC growth.

BPTES was initially identified as an inhibitor that limits excess glutamate accumulation by inhibiting the kidney-type glutaminase isoform^7^. Glucose-independent glutamine metabolism via the tricarboxylic acid (TCA) cycle has been shown to be essential for malignant cell proliferation and survival within hypoxic and nutrient-depleted tumor microenvironments^23^; this process further highlights the initial finding in which targeting glutaminase could be an effective approach for cancer therapy. Mechanistic insight has been provided in a structural study in which BPTES was shown to bind to the allosteric pocket of KGA and interfere with downstream Raf-1/Mek2/Erk signaling^24^. In this study, for the first time, we showed that glutaminase is highly expressed in both pharyngeal and oral squamous carcinoma cells (Detroit 562 and FaDu cell lines, respectively) but not in an immortalized and pre-malignant oral keratinocyte cell line (MSK-Leuk-1 cells). Analysis of the TCGA database consistently supports the prevalence of increased GLS1 expression in HNSCC patients. Our findings support a previous study in which significantly elevated GLS1 levels in oral squamous cell carcinoma (OSCC) tissues compared to healthy tissues were shown^25^. Similarly, our observation of GLS1 over-expression in HNSCC is supported in a recent paper by Kamarajan *et al*.^26^ in which it was reported that higher GLS1 expression in primary and metastatic head and neck cancer tissues compared to controls were noted. The overexpression of GLS1 is indicative of a metabolic adaption toward glutamine consumption; however, a previous study found that glutamine may not be the dominant energy source for HNSCC^27^. Of note, only FaDu cells, which express less GLS1 than Detroit 562 cells, were included in this study and glutamine utilization was not evaluated in the absence of glucose^27^. As demonstrated in our study, cell growth inhibition only became significant when a high glutamine level was supplied in the culture media. Taken together, glucose may be the dominant energy source in cells when GLS1 is not overexpressed, while glutamine may become an additional energy source once GLS1 is overexpressed and the expression of GLS1 then enables glutamine-dependent growth. Thus, it is important to determine the status of GLS1 expression in HNSCC, and it is potentially beneficial to include a GLS1 inhibitor in treatment regimens for HNSCC patients with high GLS1 levels.

Restriction of glutamine in hepatoma cells increases the levels of cellular reactive oxygen species and reduces aerobic glycolysis and pyruvate dehydrogenase kinase 2 (PDK2) expression^28^. It was suggested that the decrease in PDK2 levels was caused by p21 up-regulation in hepatoma cells^28^. In this study, we showed that p21 expression increased and the mRNA levels of several PDKs (PDK2, 3, and 4) were significantly reduced in BPTES-treated FaDu cells (Supplementary Fig. 1). It is likely that BPTES could lead to suppression of both glutaminolysis and glycolysis. Indeed, pathway analysis of our metabolomics data using MetaboAnalyst revealed that glycolysis, TCA, and nucleotide metabolism pathways were also altered in BPTES-treated Fadu cells (Supplementary Table 1).

Our data supported the hypothesis that glutamine supplementation promotes HNSCC growth, and blocking glutamine consumption using BPTES prevents HNSCC overgrowth. Therefore, GLS1 appears to be a promising therapeutic target for treating HNSCC, especially when combined with an agent that blocks glucose consumption. Of note, GLS expression does not predict the prognosis of liver cancer based on analysis of two hepatocellular carcinoma cohorts (Supplementary Fig. 2). As such, the role of GLS is likely to be specific to the context of tumor type.

Mechanistic studies revealed that BPTES caused cell cycle arrest of prostate cancer cells in the G2 phase. GLS1 induced accumulation from late G1 phase and reached a peak during the S to G2 phase^29^, indicating the key role of GLS1-mediated glutamine metabolism for intracellular biosynthesis during cell duplication. In this study, we found that BPTES dramatically up-regulated p21, a cyclin-dependent kinase inhibitor that blocks the cell cycle entering mitosis from G2 phase^30,31^. Interestingly, BPTES and metformin showed differing role in induction of cell cycle arrest. As discussed previously, cell division is an energy demanding process, requiring the production of large amounts of metabolites and biomass^32^. Although western blotting results of the cell cycle regulatory proteins could explain the restrained cell cycle phases induced by representative drugs, further investigation in clinical trials is required to understand BPTES- and metformin-induced crosstalk of metabolic pathways and potential synergy by combining the two drugs. Inhibition of GLS1 by BPTES eventually leads to apoptosis that is most likely due to energy production restriction. Indeed, there is a significant increase in the number of dead cells in the BPTES-treated HNSCC cultures.

Considering the heterogeneity and high mutation rates of the tumor tissue complex, a combined dual therapy usually achieves a better outcome than monotherapy in cancer treatment due to the dual ability to address multiple targets. Metformin is well-known for activating the 5′-adenosine monophosphate-activated protein kinase (AMPK) pathway that switches off ATP consumption, thereby inhibiting cell proliferation^33^. Metformin has been shown to induce AMPK activation and mTORC1 inhibition through the organic cation transporter 3, which is overexpressed in HNSCC^34^. In addition to the AMPK activation, metformin also targets multiple genes involved in cancer progression. Indeed, two weeks of short-term metformin treatment was shown to deliver favorable effects in HNSCC tumors through modulation of tumor cell metabolism in the HNSCC microenvironment^35^. Metformin’s pharmacological actions vary depending on its concentration^36^. High concentrations are widely used in *in vivo* and *in vitro* studies^37–39^, including this study, for the purpose of investigating metformin’s anti-cancer effects. Unlike BPTES, which causes cell cycle arrest at the S and/or G2 phase, our previous study showed that metformin could induce cell cycle arrest of salivary gland tumor cells in the S and G2 phases via inhibition of cyclin dependent kinase 1 and cyclin B^40^. In this study, metformin-induced HNSCC cell cycle arrest involved multiple different mechanisms by modulating various cell cycle regulatory proteins depending on the cell line. Metformin-treated cells accumulated in the G1 phase in FaDu cells due to inhibition of the cyclin E that transverses the G1/S checkpoint^41^; the CDK1/cyclin B2 complex was also decreased. In contrast, only the G1/S checkpoint proteins were reduced in Detroit 562 cells; thus, the cells exclusively accumulated in the S and G2 phases. Metformin is known to induce the expression of an anti-cancer miRNA, miR-708, which may facilitate inhibition of metastasis in breast cancer^25^ and promotion of apoptosis in prostate cancer^19^. Consistent with the findings in pancreatic cancer^42^, our study illustrated that the combination of BPTES and metformin provides an additive inhibitory effect on HNSCC cell proliferation.

We treated mice that had HNSCC tumors using intraperitoneal BPTES injections twice weekly for three weeks. However, tumor volume measurements showed that BPTES inhibited tumor growth, but the difference was not significant (Supplementary Fig. 3). These results may largely be due to its low solubility. At the end-point, we found crystallized BPTES in the abnormal cavity of the mice after three weeks of intraperitoneal injections. The poor solubility of BPTES could limit its efficacy *in vivo*. Using nanoparticles as a delivery vehicle to increase the uptake efficiency of BPTES^43^ could be a strategy that overcomes this obstacle. Of note, BPTES nanoparticles have been shown to be effective in a mouse model of pancreatic cancer^42^. Meanwhile, studies on structural BPTES modifications that improve its solubility in addition to screening BPTES analogs for stronger bioactivity should be pursued.

Taken together, this study confirms the efficacy of interfering with the cancer-associated glutamine consumption as a potential therapy in HNSCC via induction of cell cycle arrest and apoptosis. The additive effects of combined treatment of BPTES and metformin in HNSCC warrants further investigation in preclinical models and clinical trials.

## Materials and Methods

### Cell culture

The human pharynx squamous cancer and human oral leukoplakia cell lines, FaDu and MSK-Leuk-1, respectively, were obtained from Peter Sacks (New York University College of Dentistry). The human pharyngeal carcinoma cell line Detroit 562 (derived from a pleural effusion) was purchased from the American Type Culture Collection. FaDu and Detroit 562 cells were cultured in Minimum Essential Medium (MEM) Alpha Medium (Corning Inc. Corning, NY, USA) supplemented with 10% fetal bovine serum (Atlanta Biologicals, Flowery Branch, GA, USA) and 1% penicillin-streptomycin (Sigma, St. Louis, MO, USA). All cells were maintained in a 37 °C, 5% (v/v) CO_2_ growth chamber (Sanyo, Moriguchi, Japan).

### TCGA data analysis

The GLS1 expression profile data originally released from the TCGA were downloaded from cBioportal (http://www.cbioportal.org/). A total of 44 healthy oral samples and 522 HNSCC samples were included in this study. GLS1 expression levels of all individual samples were extracted using an R program. Graphs comparing GLS1 expressions in healthy and tumorigenic samples and the disease-free curves of the GLS-high and GLS-low HNSCC subgroups were generated using Prism GraphPad software (GraphPad Software, San Diego, CA).

### Western blotting

Anti-human GLS1 and cyclin B1 primary antibodies were purchased from Novus Biologicals Inc. (Littleton, CO, USA). Anti-human caspase-3, cleaved-caspase-3, poly (adenosine phosphate ribose) polymerase (PARP), cleaved-PARP, CDK1, cyclins B1 and E2, p21, glyceraldehyde 3-phosphate dehydrogenase, and β-actin primary antibodies in addition to all horseradish peroxidase secondary antibodies were purchased from Cell Signaling Technology (Danvers, MA, USA). The protein samples were preheated with the sodium dodecyl sulfate sample buffer and NuPAGE reducing buffer at 85 °C for 2 min and then loaded onto a Tris-glycine gel (Invitrogen, Carlsbad, CA, USA). Electrophoresis was performed in a Mini Gel tank (Life Technologies, Carlsbad, CA, USA). Protein samples were transferred to a polyvinylidene difluoride membrane for primary antibody overnight incubation at 4 °C. A SuperSignal West Dura Extended Duration Substrate kit (Thermo Scientific, Rockford, IL, USA) was used for signal detection and imaging.

### Flow cytometry

The cells were cultured in six-well plates and treated with BPTES (SelleckChem, Houston, TX, USA) or metformin (MP Biomedicals, Solon, OH, USA) for 48 h. After trypsinization and fixation, the cells were prepared for flow cytometry. For apoptosis assays, the cells were stained with PI and annexin V using the FITC Annexin V Apoptosis Detection kit (BD Biosciences, Franklin Lakes, NJ, USA). For cell cycle assays, the cells were stained with a PI PI/Triton X-100 staining solution containing 0.1% (v/v) Triton X-100 (EM Science, Darmstadt, Germany), 0.2 mg/ml DNAse-free RNAse A (Thermo Scientific), and 20 mg/ml PI (Roche, Basel, Switzerland) for 15 minutes at 37 °C. Flow cytometry was performed on a BD FACSCalibur analyzer (BD Biosciences). The data were interpreted using the FlowJo software.

### Crystal violet staining

The cells were fixed with 3.7% paraformaldehyde (Fisher Scientific, Kalamazoo, MI, USA) for 5 min and then stained with 0.05% crystal violet (Sigma) for 30 min. After staining, the cells were washed with tap water twice to remove any residual dye and then drained for a couple of minutes. One third to one half of the total well volume of methanol was added in order to solubilize the dye by incubating on a shaker for 5 min. The relative cell numbers were determined by reading the absorbance at wavelength 540.

### MTT assay

MTT powder (Life Technologies) was dissolved in phosphate-buffered saline ([PBS]; 5 mg/ml stock solution). The cells were seeded in a 96-well plate. After treatment with BPTES or metformin for 48 h, the medium was replaced with the MTT stock mixed with fresh culture medium (1:10). The cells were then cultured at 37 °C for 4 h. After labeling the cells with MTT, all but 25 μl of the medium was removed and 50 μl of dimethyl sulfoxide was added to dissolve the dye. The relative cell viability was determined by reading the absorbance at OD 540.

### Study approval

All animal procedures were approved by the Institutional Animal Care and Use Committee (IACUC) at New York University Medical Center and were in compliance with ethical regulations.

### Statistics

All of the data are displayed as mean ± SEM. The unpaired *t*-test was used for the comparison of two groups. A one-way analysis of variance was applied when needed. A Kaplan-Meier survival curve was generated in order to represent the difference between the GLS-low and GLS-high subgroups of the TCGA dataset using the GraphPad Instat Software Program (GraphPad Software, San Diego, CA). A p-value < 0.05 was considered to be statistically significant.

## Supplementary information

SUPPLEMENTARY INFO

## Data Availability

All the raw data are available upon request. The metabolomics raw dataset will be uploaded to Metabolomics Workbench for free public access.
