# Supplementary material for: Targeting cellular metabolism to reduce head and neck cancer growth
Source: Sci Rep. 2019 Mar 21;9:4995. doi: 10.1038/s41598-019-41523-4 (PMC6428890; doi:10.1038/s41598-019-41523-4)
Supplement: Supplementary file 1 — SUPPLEMENTARY INFO [file 41598_2019_41523_MOESM1_ESM.pdf]

Targeting cellular metabolism to reduce head and neck cancer growth

Jian Yang[1], Yuqi Guo[1], Wonkyu Seo[1], Ruohan Zhang[1], Cuijie Lu[1], Yaoyu Wang[1], Liang Luo[1], Bidisha Paul[1], Wenbo Yan[1], Deepak Saxena[1,2], Xin Li[1,3,4*]

[1] Department of Basic Science and Craniofacial Biology, New York University College of Dentistry, New York, NY 10010, [2] Department of Surgery, [3] Department of Urology, [3]Perlmutter Cancer Institute, New York University Langone Medical Center, New York, NY 10016.

*To whom correspondence should be addressed:
Xin Li, Ph.D

Associate Professor

Department of Basic Science and Craniofacial Biology

New York University-College of Dentistry

345 East 24th street, Room 901D,

New York, NY, 10010

xl15@nyu.edu

S Table 1

Significantly altered metabolism pathways in BPTES treated Fadu cells.

Pathways	Total	Hits	Raw p	FDR
**Alanine, aspartate and glutamate metabolism**	24	5	1.07E-05	0.000854
**Pyrimidine metabolism**	60	6	0.000102	0.004077
**D-Glutamine and D-glutamate metabolism**	11	3	0.000328	0.008227
**Arginine and proline metabolism**	77	6	0.000411	0.008227
**Purine metabolism**	92	6	0.001074	0.017182
**Nitrogen metabolism**	39	4	0.001528	0.020374
**Citrate cycle (TCA cycle)**	20	3	0.002088	0.023861
**Glycolysis or Gluconeogenesis**	31	3	0.007454	0.074541

Pathway analysis of metabolomics data (same as Fig 1F) using MetaboAnalyst.

S Figure 1

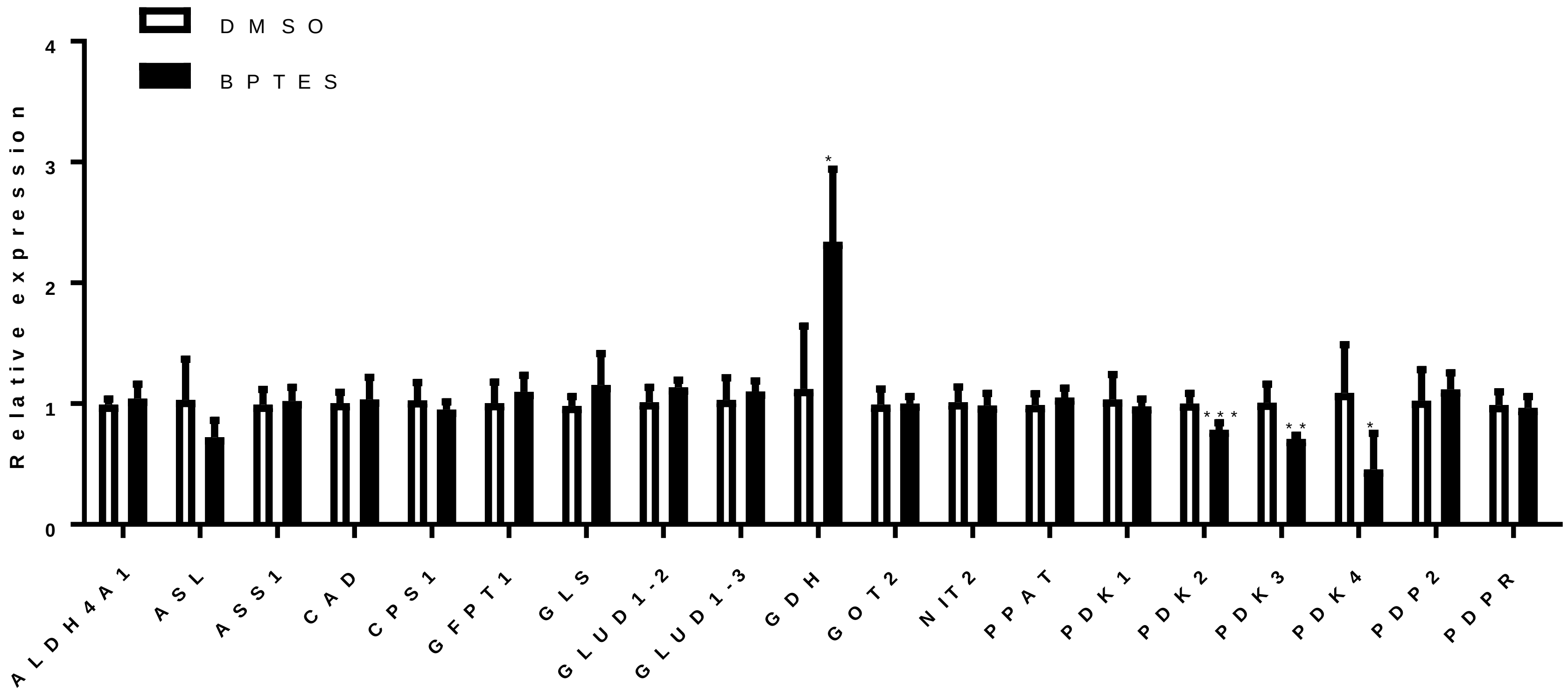
DMSO
BPTES
Relative expression
0
1
2
3
4
*
***
**
*
ALDH4A1
ASL
ASS1
CAD
CPS1
GFPT1
GLS
GLUD1-2
GLUD1-3
GDH
GOT2
NIT2
PPAT
PDK1
PDK2
PDK3
PDK4
PDP2
PDPR

S Figure 2

A

Cohort 1

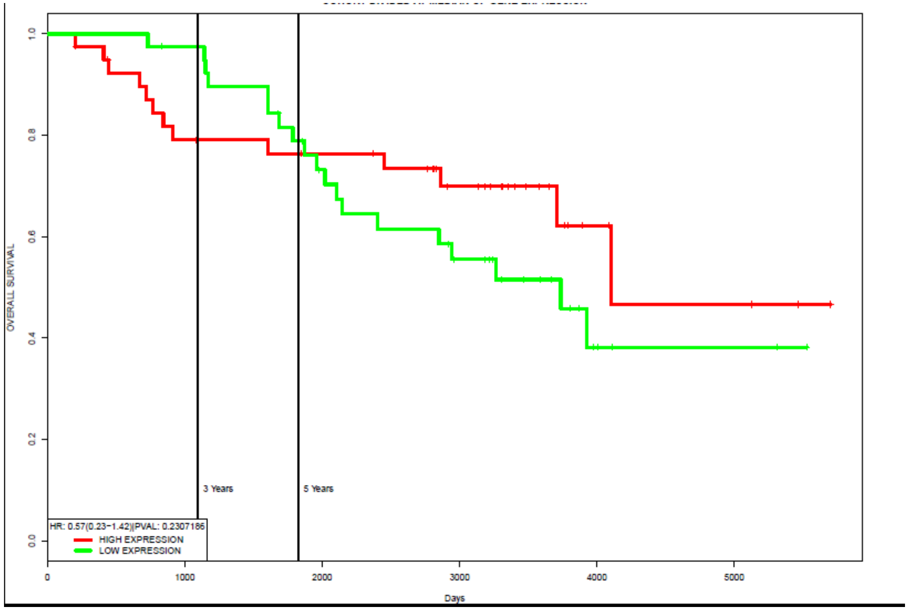

HAZARD RATIO	LCI (95%)	UCI (95%)	P VALUE
0.57	0.23	1.42	0.230718609611731

CATEGORY	SAMPLES	NO OF EVENTS	MEDIAN SURVIVAL	LOW CONF INT (95%)	UPP CONF INT (95%)
HIGH	40	13	4105	3708	NA
LOW	40	19	3736	2405	NA

B

Cohort 2

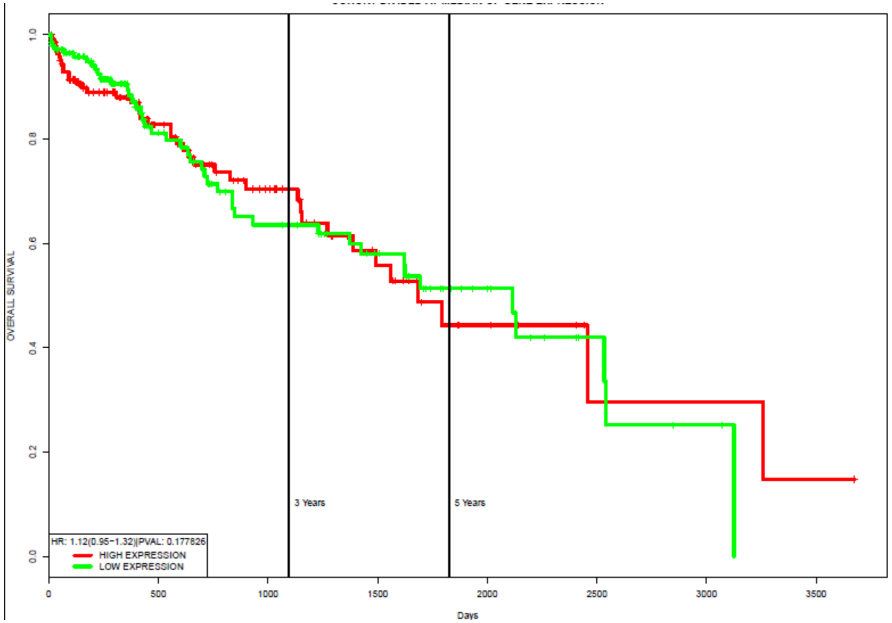

HAZARD RATIO	LCI (95%)	UCI (95%)	P VALUE
1.12	0.95	1.32	0.177825998195408

CATEGORY	SAMPLES	NO OF EVENTS	MEDIAN SURVIVAL	LOW CONF INT (95%)	UPP CONF INT (95%)
HIGH	147	41	1685	1386	NA
LOW	146	43	2116	1372	NA

S Figure 3

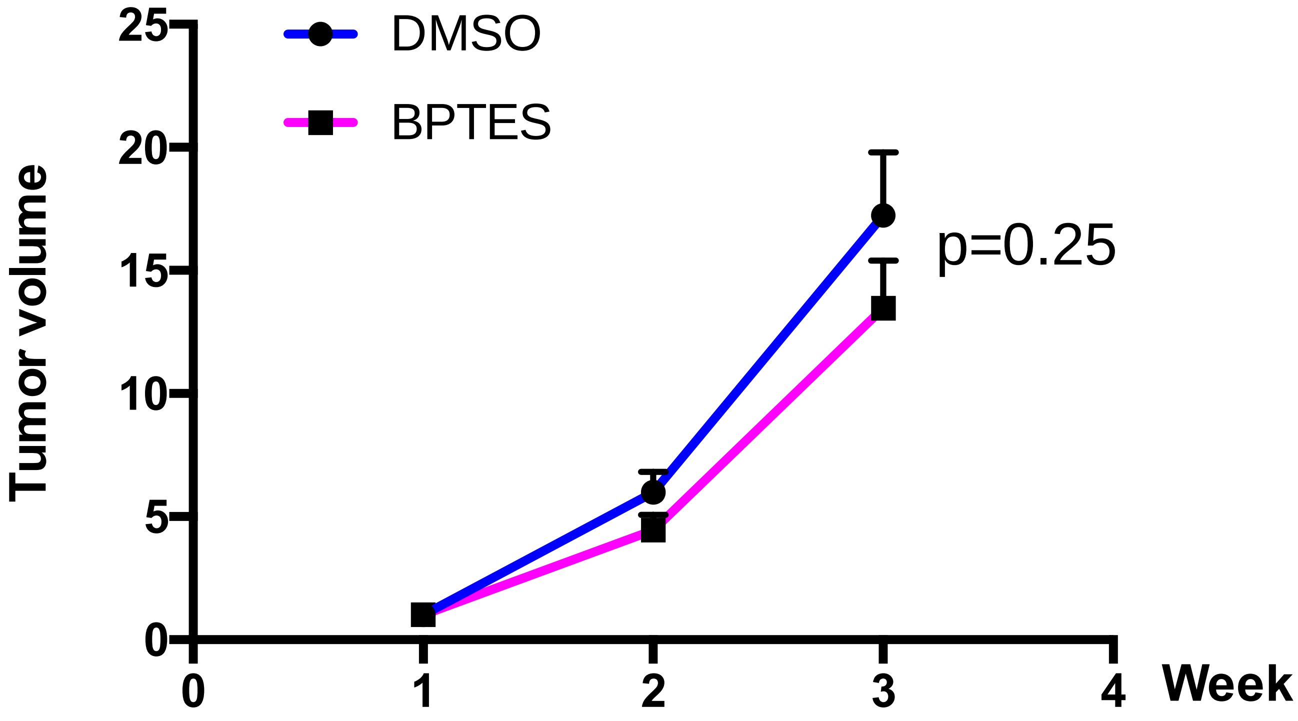

Supplementary figure 1: Regulatory effect of BPETS on metabolic enzymes. FaDu cells was treated with BPTES (10 uM) for 72 hours, DMSO was used as vehicle control. Relative expression of indicated genes was evaluated by qPCR. *p <0.05, **p<0.01, *** p<0.001,

Supplementary figure 2: Overall survival of 2 cohorts of liver cancer patients with high or low expression of GLS. The data was obtained from ProgGeneV2 database (http://watson.compbio.iupui.edu/chirayu/proggene/database/?url=proggene).

Supplementary figure 3: The effect of BPTES on HNSCC tumor growth. Twenty athymic nude mice were assigned to two groups randomly. Each athymic nude mouse was inoculated with a half million of FaDu cells subcutaneously. DMSO solution (10% DMSO in PBS) at 200 μl volume or BPTES solution (200 μg BPTES in 200 μl DMSO solution, 8mg/kg per mouse) was intraperitoneally injected into the mice twice per week for three weeks. The tumor volumes were measured weekly using vernier calipers and calculated using the following formula: length (mm) × width (mm) × width (mm) × 0.52 (Xiang et al., 2015).

Xiang Y, Stine ZE, Xia J, Lu Y, O'Connor RS, Altman BJ *et al.* (2015). Targeted inhibition of tumor-specific glutaminase diminishes cell-autonomous tumorigenesis. *The Journal of clinical investigation* 125(6):2293-2306.
